# Supplementary material for: Identification of metabolites associated with preserved muscle volume after aneurysmal subarachnoid hemorrhage due to high protein supplementation and neuromuscular electrical stimulation
Source: Sci Rep. 2024 Jul 2;14:15071. doi: 10.1038/s41598-024-64666-5 (PMC11219968; doi:10.1038/s41598-024-64666-5)
Supplement: Supplementary file 1 — Supplementary Information. [file 41598_2024_64666_MOESM1_ESM.docx]

**Supplementary Material**

Supplementary Figure 1: Boxplots of protein per day, nitrogen balance, and muscle volumes.

Supplementary Figure 2: Classes of metabolites detected.

Supplementary Table 1: Differentially expressed metabolites.

Supplementary Table 2: Metabolites identified from sPLS-DA analysis.

Supplementary Figure 3: Boxplots of changes in key metabolites comparing SOC and HPRO+NMES groups.

Supplementary Table 3: Correlations between metabolites and protein intake per day and nitrogen balance.

Supplementary Table 4: Correlations between metabolites and preservation of temporalis and quadricep muscle mass.

Supplementary Table 5: Classification and function of relevant metabolites


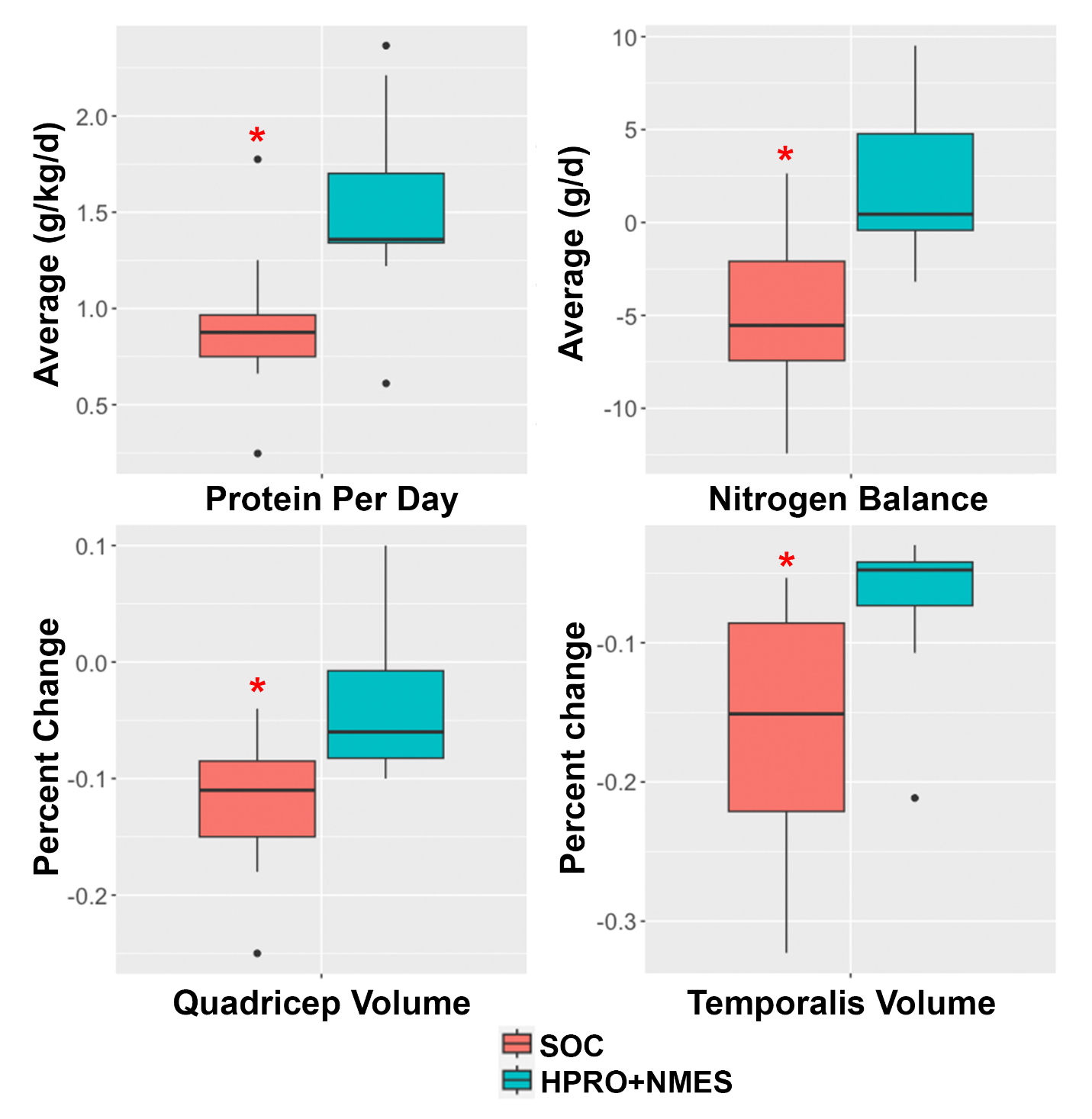


Supplementary Figure 1: Average protein intake (protein per day), nitrogen balance, and percent change in muscle (quadricep and temporalis) volume are presented as boxplots comparing the two groups (SOC vs HPRO+NMES). **p*<0.05. Abbreviations: standard of care (SOC), high protein diet combined with neuromuscular electrical stimulation (HPRO+NMES).


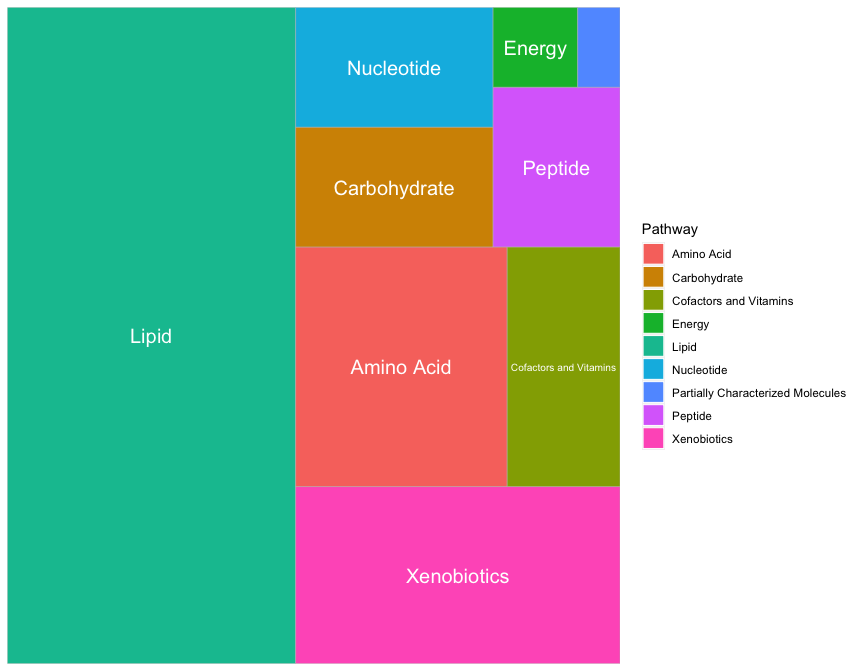


Supplementary Figure 2: Tree diagram depicting classes of metabolites detected. 1,109 metabolites were detected, with 261 unable to be fully characterized.

| **Metabolite** | **log2(Fold Change)** | **P-Value** | **FDR P-Value** |
| --- | --- | --- | --- |
| 6-oxopiperidine-2-carboxylate | 11.6 | 0.00005360 | 0.030422 |
| beta-hydroxyisovaleroylcarnitine | 10.1 | 0.00003370 | 0.030422 |
| N-acetylkynurenine | 10.3 | 0.00008910 | 0.030422 |
| N-acetylvaline | 10.3 | 0.00023081 | 0.037708 |
| picolinoylglycine | 9.68 | 0.00009310 | 0.030422 |
| quinolinate | 7.87 | 0.00018595 | 0.034719 |
| tiglyl carnitine | 9.87 | 0.00017910 | 0.034719 |
| urea | 9.91 | 0.00029023 | 0.042147 |
| 3-CMPFP | -5.14 | 0.00011689 | 0.030556 |

Supplementary Table 1: Significantly increased or decreased metabolites in paired analysis consider pre and post randomization timepoints in the HPRO+NMES group.

| **Comparison** | **Metabolite** | **comp 1** |
| --- | --- | --- |
| HRPO+NMES Paired Early vs Late | β-hydroxyisovaleroylcarnitine | -0.49812 |
|  | 6-oxopiperidine-2-carboxylate | -0.43525 |
|  | N-acetylkynurenine (2) | -0.36185 |
|  | picolinoylglycine | -0.35535 |
|  | 3-carboxy-4-methyl-5-pentyl-2-furanpropionate (3-CMPFP) | 0.32084 |
|  | tiglyl carnitine (C5) | -0.25341 |
|  | quinolinate | -0.2473 |
|  | N-acetylvaline | -0.21155 |
|  | urea | -0.17255 |
|  | butyrylglycine (C4) | -0.048931 |
|  |  |  |
| Change in Metabolites SOC vs HPRO+NMES | β-hydroxyisovaleroylcarnitine | 0.68271 |
|  | N-acetylleucine | 0.50979 |
|  | N-acetylserine | 0.3605 |
|  | N-acetylvaline | 0.2795 |
|  | N-acetylisoleucine | 0.19039 |
|  | 3-phosphoglycerate | 0.16744 |
|  | genistein sulfate | -0.030324 |
|  | daidzein sulfate | -0.019199 |
|  | N-acetylthreonine | 0.016075 |
|  | 1-oleoyl-2-arachidonoyl-GPE (18:1/20:4) | 0.010125 |

Supplementary Table 2: Primary metabolites identified in sPLS-DA analysis to account for differences between groups. Comparisons include changes in metabolites in the HPRO+NMES before randomization and at day 7 after the protocol as well as differences in metabolites comparing the SOC and HPRO+NMES group. A total of 18 unique metabolites were identified. Abbreviations: standard of care (SOC), high protein diet combined with neuromuscular electrical stimulation (HPRO+NMES).


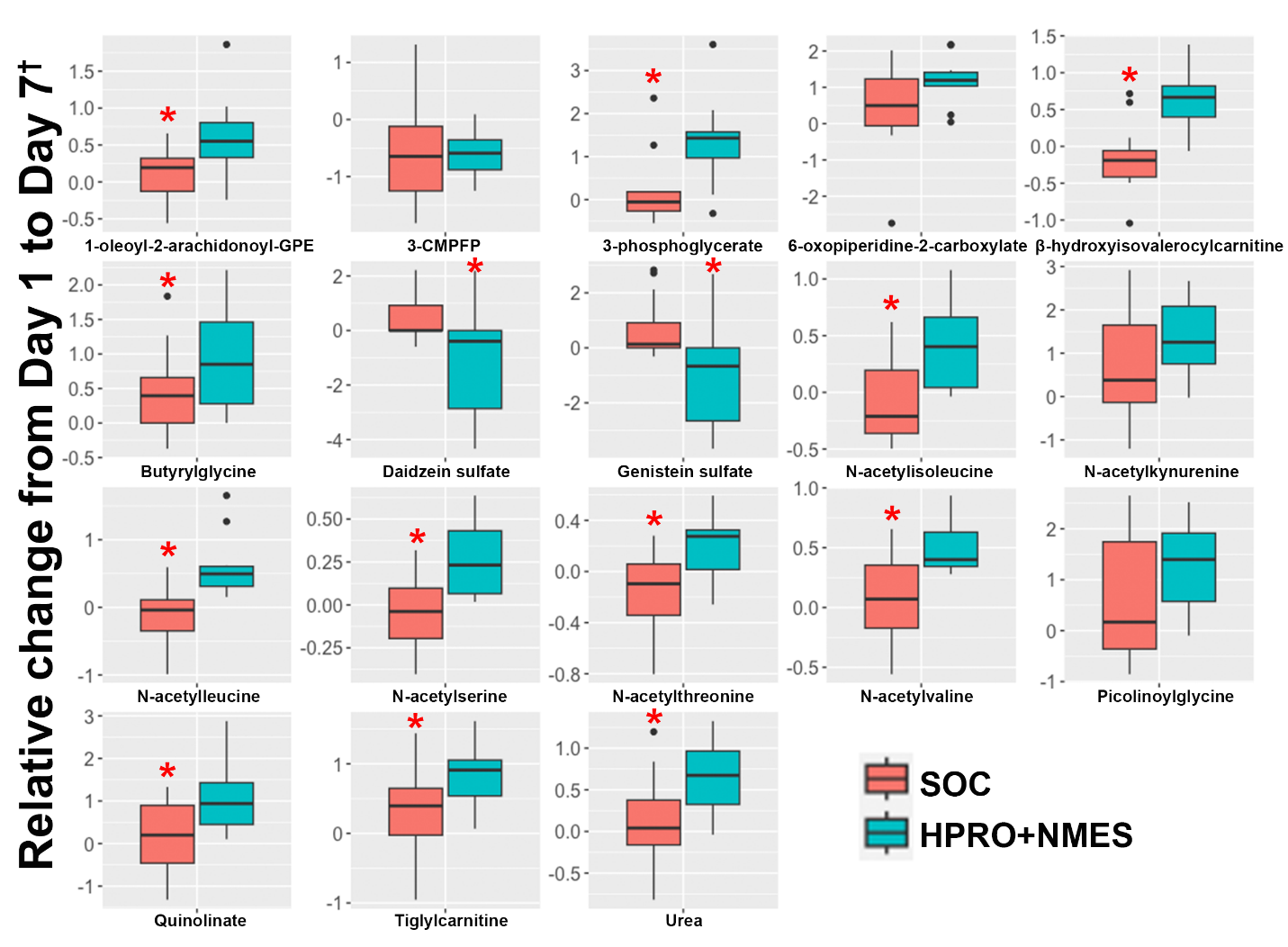


Supplementary Figure 3: Changes in metabolites from baseline to day 7 comparing standard of care (SOC) and high protein + neuromuscular electrical stimulation (HPRO+NMES). Data are presented for each of the 18 metabolites identified in Supplementary Table 2. Values on the y-axis represent changes in relative levels (areas under the curve) for each metabolite from baseline (day 1, pre-randomization) to day 7. **p*<0.05.

|  |  | Protein Intake Per Day | | Nitrogen Balance | |
| --- | --- | --- | --- | --- | --- |
|  |  | ρ | *P* | ρ | *P* |
| 18 Metabolites identified in association with HPRO+NMES intervention | **N-acetylserine** | **0.610** | **1.56x10^-3^** | **0.530** | **9.288x10^-3^** |
|  | **N-acetylleucine** | **0.580** | **2.97x10^-3^** | **0.654** | **7.20x10^-4^** |
|  | **β-hydroxyisovalerocylcarnitine** | **0.526** | **8.35x10^-3^** | **0.490** | **0.0175** |
|  | Tiglylcarnitine | 0.483 | 0.0168 | 0.356 | 0.0954 |
|  | N-acetylisoleucine | 0.477 | 0.0183 | 0.318 | 0.139 |
|  | **N-acetylthreonine** | **0.466** | **0.0218** | **0.579** | **3.79x10^-3^** |
|  | N-acetylkynurenine | 0.453 | 0.0263 | 0.252 | 0.246 |
|  | N-acetylvaline | 0.442 | 0.0306 | 0.248 | 0.254 |
|  | Urea | 0.426 | 0.0381 | 0.326 | 0.129 |
|  | Quinolinate | 0.384 | 0.0637 | 0.521 | 0.0108 |
|  | Picolinoylglycine | 0.365 | 0.0795 | 0.119 | 0.588 |
|  | 6-oxopiperidine-2-carboxylate | 0.344 | 0.0995 | 0.317 | 0.140 |
|  | 1-oleoyl-2-arachidonoyl-GPE (18:1/20:4) | 0.318 | 0.131 | 0.457 | 0.0284 |
|  | 3-phosphoglycerate | 0.156 | 0.468 | 0.0446 | 0.840 |
|  | 3-CMPFP | 0.0758 | 0.725 | -0.129 | 0.557 |
|  | Butyrylglycine | 0.0315 | 0.884 | 0.0422 | 0.848 |
|  | Daidzein sulfate | -0.255 | 0.230 | -0.177 | 0.419 |
|  | Genistein sulfate | -0.265 | 0.210 | -0.137 | 0.534 |
|  | | | | | |
| Metabolites with negative correlations with Protein intake | hexadecenedioate (C16:1-DC) | -0.658 | 4.71x10^-4^ | -0.387 | 0.0683 |
|  | **tetradecadienedioate (C14:2-DC)** | **-0.645** | **6.67x10^-4^** | **-0.565** | **4.97x10^-3^** |
|  | **3-hydroxydodecanedioate** | **-0.595** | **2.14x10^-3^** | **-0.455** | **0.0292** |
|  | 3-hydroxybutyrate (BHBA) | -0.576 | 3.22x10^-3^ | -0.357 | 0.0948 |
|  | acetoacetate | -0.568 | 3.78x10^-3^ | -0.330 | 0.124 |
|  | **tetradecanedioate (C14)** | **-0.535** | **7.12x10^-3^** | **-0.466** | **0.0249** |
|  | octadecanedioate (C18) | -0.527 | 8.21x10^-3^ | -0.316 | 0.141 |
|  | hexadecanedioate (C16) | -0.522 | 8.89x10^-3^ | -0.337 | 0.115 |
|  | dodecanedioate (C12) | -0.508 | 0.0113 | -0.391 | 0.0652 |

Supplementary Table 3: Correlations between metabolites and protein intake per day and nitrogen balance. Bolded metabolites have significant correlations between both protein intake per day and nitrogen balance.

|  |  | Temporalis Volume | | Quadricep  Volume | |
| --- | --- | --- | --- | --- | --- |
|  |  | ρ | *P* | ρ | *P* |
| 18 Metabolites identified in association with HPRO+NMES intervention | **Nitrogen Balance** | **0.643** | **0.00124** | **0.433** | **0.0442** |
|  | **Protein per day** | **0.634** | **0.00152** | **0.623** | **0.00197** |
|  | N-acetylisoleucine | 0.527 | 0.0117 | 0.377 | 0.0834 |
|  | N-acetylserine | 0.502 | 0.0174 | 0.378 | 0.0827 |
|  | **N-acetylleucine** | **0.471** | **0.0270** | **0.535** | **0.0102** |
|  | **Quinolinate** | **0.447** | **0.0371** | **0.355** | **0.105** |
|  | N-acetylvaline | 0.415 | 0.0546 | 0.409 | 0.0589 |
|  | N-acetylthreonine | 0.407 | 0.0604 | 0.298 | 0.177 |
|  | Tiglylcarnitine | 0.405 | 0.0616 | 0.168 | 0.454 |
|  | β-hydroxyisovalerocylcarnitine | 0.402 | 0.0635 | 0.444 | 0.0384 |
|  | Urea | 0.358 | 0.102 | 0.280 | 0.206 |
|  | Daidzein sulfate | -0.320 | 0.146 | -0.216 | 0.333 |
|  | Genistein sulfate | -0.281 | 0.205 | -0.238 | 0.285 |
|  | Butyrylglycine | 0.255 | 0.252 | 0.145 | 0.519 |
|  | N-acetylkynurenine | 0.199 | 0.375 | 0.247 | 0.267 |
|  | 6-oxopiperidine-2-carboxylate | 0.130 | 0.564 | 0.132 | 0.557 |
|  | Picolinoylglycine | 0.0894 | 0.692 | 0.179 | 0.424 |
|  | 1-oleoyl-2-arachidonoyl-GPE (18:1/20:4) | 0.0857 | 0.705 | 0.384 | 0.0769 |
|  | 3-CMPF | 0.0277 | 0.903 | 0.119 | 0.597 |
|  | 3-phosphoglycerate | 0.0234 | 0.918 | 0.277 | 0.212 |
|  | | | | | |
| Metabolites with negative correlations with Protein intake | hexadecenedioate (C16:1-DC) | -0.348 | 0.113 | -0.420 | 0.0517 |
|  | tetradecadienedioate (C14:2-DC) | -0.417 | 0.0533 | -0.446 | 0.0374 |
|  | 3-hydroxydodecanedioate | -0.278 | 0.211 | -0.483 | 0.0228 |
|  | 3-hydroxybutyrate (BHBA) | -0.319 | 0.148 | -0.451 | 0.0350 |
|  | acetoacetate | -0.280 | 0.206 | -0.240 | 0.281 |
|  | tetradecanedioate (C14) | -0.333 | 0.130 | -0.488 | 0.0214 |
|  | octadecanedioate (C18) | -0.224 | 0.316 | -0.340 | 0.122 |
|  | hexadecanedioate (C16) | -0.221 | 0.322 | -0.384 | 0.0778 |
|  | dodecanedioate (C12) | -0.267 | 0.230 | -0.347 | 0.114 |

Supplementary Table 4: Correlations between metabolites and preservation of temporalis and quadriceps muscle volume. Bolded metabolites and parameters had significant correlations with preservation of both temporalis and quadricep muscles.

| Metabolite | Category | Biological Function |
| --- | --- | --- |
| N-acetylserine | N-Acyl amino acids | Amino acid metabolism, various signaling pathways^1,2^ |
| N-acetylleucine |  |  |
| N-acetylisoleucine |  |  |
| N-acetylthreonine |  |  |
| N-acetylkynurenine |  |  |
| N-acetylvaline |  |  |
| Picolinoylglycine |  |  |
| β-hydroxyisovalerocylcarnitine | Acylcarnitines | Energy source, markers of altered metabolism^3^ |
| Tiglylcarnitine |  |  |
| Butyrylglycine | Acylglycines | Glycine metabolism, anti-inflammatory, modulation of vascular tone^4^ |
| Urea | Carbamide | Ammonia metabolism^5^ |
| Quinolinate | Pyridinecarboxylic acids | Kynurenine pathway of tryptophan catabolism, NADH/NAD+ generation^6^ |
| 6-oxopiperidine-2-carboxylate | Monocarboxylic acid | Lysine metabolism^7^ |
| 1-oleoyl-2-arachidonoyl-GPE (18:1/20:4) | Glycerophospholipid | Cell membrane stability, fluidity, and permeability^8^ |
| 3-phosphoglycerate | Monophosphoglycerate | Intermediate in glycolysis and Calvin cycle^9^ |
| 3-CMPFP | Furanoid fatty acid | Uremic toxin |
| Daidzein sulfate | Isoflavones | Bioactive phytochemical, modification of inflammatory cell function^10^ |
| Genistein sulfate |  |  |

Supplementary Table 5: Summary of the classification and biological function of each of the 18 metabolites identified to be increased due to the HPRO+NMES treatment.

**References**

1. Sarkar, C. & Lipinski, M. M. N-acetyl-L-leucine: a promising treatment option for traumatic brain injury. *Neural Regen Res* **17**, 1957–1958 (2022).

2. Morales-Tarré, O., Alonso-Bastida, R., Arcos-Encarnación, B., Pérez-Martínez, L. & Encarnación-Guevara, S. Protein lysine acetylation and its role in different human pathologies: a proteomic approach. *Expert Rev Proteomics* **18**, 949–975 (2021).

3. Dambrova, M. *et al.* Acylcarnitines: Nomenclature, Biomarkers, Therapeutic Potential, Drug Targets, and Clinical Trials. *Pharmacol Rev* **74**, 506–551 (2022).

4. Anderson, R. L. & Merkler, D. J. N-FATTY ACYLGLYCINES: UNDERAPPRECIATED ENDOCANNABINOID-LIKE FATTY ACID AMIDES? *J Biol Nat* **8**, 156–165 (2017).

5. Weiner, I. D., Mitch, W. E. & Sands, J. M. Urea and Ammonia Metabolism and the Control of Renal Nitrogen Excretion. *Clin J Am Soc Nephrol* **10**, 1444–58 (2015).

6. Moffett, J. R. *et al.* Quinolinate as a Marker for Kynurenine Metabolite Formation and the Unresolved Question of NAD+ Synthesis During Inflammation and Infection. *Front Immunol* **11**, 31 (2020).

7. Kuan, P.-F. *et al.* Metabolomics analysis of post-traumatic stress disorder symptoms in World Trade Center responders. *Transl Psychiatry* **12**, 174 (2022).

8. Hishikawa, D., Hashidate, T., Shimizu, T. & Shindou, H. Diversity and function of membrane glycerophospholipids generated by the remodeling pathway in mammalian cells. *J Lipid Res* **55**, 799–807 (2014).

9. Le Moigne, T. *et al.* Photoproduction of reducing power and the Calvin-Benson cycle. in *The Chlamydomonas Sourcebook* 273–315 (Elsevier, 2023). doi:10.1016/B978-0-12-821430-5.00016-X.

10. Mace, T. A. *et al.* Soy isoflavones and their metabolites modulate cytokine-induced natural killer cell function. *Sci Rep* **9**, 5068 (2019).
